# Establishment and application of quadruple fluorescence quantitative RT-PCR method for the identification of waterfowl astrovirus

**DOI:** 10.3389/fmicb.2024.1328243

**Published:** 2024-07-10

**Authors:** Yang Li, Juan Luo, Fuyou Zhang, Jiajing Shang, Chunran Deng, Yingjie Feng, Ge Meng, Wenming Jiang, Xiaohui Yu, Guanhui Liu, Hualei Liu

**Affiliations:** ^1^China Animal Health and Epidemiology Center, Qingdao, Shandong, China; ^2^College of Life Sciences and Food Engineering, Hebei University of Engineering, Handan, Hebei, China

**Keywords:** DAstV-3, DAstV-4, GoAstV-1, GoAstV-2, quadruple fluorescence quantitative RT-PCR

## Abstract

Avian astrovirus can infect a variety of poultry species and cause viral diarrhea, with a wide epidemic range strong pathogenicity and a high incidence. Among them, Duck astrovirus 3(DAstV-3), Duck astrovirus 4(DAstV-4), Goose astrovirus 1(GoAstV-1) and Goose astrovirus 2(GoAstV-2) are four types of astroviruses newly discovered in waterfowl in recent years. In order to realize the rapid detection of these four kinds of waterfowl stellate viruses, specific primers and probes were engineered to target a highly conserved region of ORF1b gene of DAstV-3, GoAstV-1 and GoAstV-2 and the ORF2 gene of DAstV-4, and a quadruple fluorescence quantitative RT-PCR method was developed. The results indicate that the method established in this study has good specificity and no cross reactivity with other pathogens. This method can detect viruses with a minimum concentration of 1 × 10^1^ copies/μL for DAstV-4, GoAstV-1 and GoAstV-2, respectively, while the minimum concentration for DAstV-3 is 1 × 10^2^ copies/μL. Compared with the routinely used RT-PCR method, the limit of detection by the multiplex RT-PCR lower. Both intra- and inter-assay variability tests revealed excellent reproducibility. This method was then used to analyze 269 field samples, and the results were verified by genome sequencing. In conclusion, this study presents a sensitive, accurate, and specific method for detecting DAstV-3, DAstV-4, GoAstV-1, and GoAstV-2 in a single reaction, enabling the monitoring and differential diagnosis of these four types of waterfowl astroviruses.

## 1 Introduction

Astroviruses are a class of small, non-enveloped virus with a single-stranded RNA that belong to the family *Astroviridae*. This family contains two genera: Mamastroviruses (MAstVs) and Avastroviruses (AAstVs) according to the different types of infected hosts (Koci and Schultz-Cherry, 2002). The genus *Mamastrovirus* includes astrovirus species that have been isolated from humans and a variety of mammals, such as porcine astrovirus, mink astrovirus, bovine astrovirus, and canine astrovirus. *Avastrovirus* includes astrovirus species that have been isolated from poultry, such as Turkey astrovirus, Chicken astrovirus, Avian nephritis virus, Duck astrovirus, and Goose astrovirus, etc. (De Benedictis et al., 2011; Chen et al., 2021). In addition, some astroviruses have not been classified. The genetic evolution of astroviruses shows that astroviruses have the potential to cross species barriers and adapt to new hosts (Roach and Langlois, 2021). The genome length of the virus is 6.8~7.9 kb, consisting of a 5′ untranslated region (UTR), three open reading frames (ORFs)-ORF1a, ORF1b, and ORF2, a 3′ UTR, and a poly-A tail. ORF1a and ORF1b are highly conserved regions that code for nonstructural proteins. ORF1b encodes RNA dependent RNA polymerase (RDRP) while ORF2 encodes the viral capsid protein. ORF2 is a variable region that interacts specifically with virus-specific antibodies and helps in determining phylogenetic relationships between viruses (Strain et al., 2008). The research of the Astrovirus Research Group of the International Committee of Taxonomy of Viruses (ICTV) shows that the classification of viruses is generally based on the genetic distance between the host and the whole amino acid sequence of the structural protein, but the viruses with more than 75% homology in the whole gene sequence of ORF2 protein should be considered as members of the same genotype (Tan et al., 2023).

Avian astrovirus can infect a variety of avian species and cause intestinal and other internal organ diseases, such as broiler dysplasia syndrome, gosling gout infection, duck viral hepatitis, viral diarrhea (Chu et al., 2012; Donato and Vijaykrishna, 2017; Niu et al., 2018). At present, the reported waterfowl astrovirus mainly includes six types, Duck astrovirus 1 (DAstV-1), DAstV-2, DAstV-3, DAstV-4, Goose astrovirus 1 (GoAstV-1) and GoAstV-2. DAstV-1 and DAstV-2 have been officially classified by the ICTV as astroviruses and the main symptom of infection being duck hepatitis, which has a rapid onset (Yugo et al., 2016; Wei et al., 2022). DAstV-3 was first reported in Peking ducks in 2013. In recent years, it has been widely spread with high infection rate and morbidity, which is harmful to ducklings and has a certain effect on duck embryo hatching, leading to serious damage to the chorioallantoic membrane, growth retardation and embryo death (Liu et al., 2014; Li et al., 2021). There were few reports about DAstV-4, and its pathogenicity needs further investigation (Liao et al., 2015). GoAstV-1 causes urate deposition in the liver, ureters, kidneys, joints, and other internal organs, leading to swelling of the kidneys and liver (Zhang et al., 2022). GoAstV-2 is a serious infectious disease found in 2016–2018, which is characterized by gout, hemorrhage and swelling of the kidneys, accompanied by urate deposits, white feces and paralysis (Chen et al., 2020; Zhu et al., 2022). Among them, waterfowl-derived astroviruses, such as DAstV-3, DAstV-4, GoAstV-1, and GoAstV-2, were newly discovered over recent years, which bring great harm to poultry industry and there were no effective biological agents to prevent and control. Therefore, accurate detection method is very important for the diagnosis, prevention and control of this disease.

At present, the detection methods for waterfowl astrovirus include virus isolation, RT-PCR (Reverse Transcription-Polymerase Chain Reaction), fluorescence quantitative RT-PCR, Loop-mediated isothermal amplification (LAMP), and so on (Liu et al., 2016; Yu et al., 2020; Wang et al., 2022). Virus isolation, RT-RT-PCR take a long time and are not suitable for rapid clinical detection. The LAMP technology takes a short time and can realize macroscopic observation, but is prone to false positive results. On the other hand, fluorescent quantitative RT-PCR has the advantages of simple operation, fewer steps, no need for agarose gel electrophoresis, and the lab capability of avoiding contamination to a certain extent; and simultaneously, the multiple fluorescent quantitative RT-PCR method simultaneously detects a plurality of viruses and realizes the detection of a plurality of pathogens.

This study intends to develop a rapid, sensitive and specific quadruple fluorescence quantitative RT-PCR method simultaneous detection of DAstV-3, DAstV-4, GoAstV-1 and GoAstV-2, and provides technical support for epidemiological investigation and prevention and control of waterfowl-derived astrovirus.

## 2 Materials methods

### 2.1 Virus and clinical samples

The DAstV-3, DAstV-4, GoAstV-1, GoAstV-2, Newcastle disease virus (NDV), H9 subtype avian influenza Virus (H9-AIV), Avian reovirus (ARV) were isolated and stored in the China Animal Health and Epidemiology Center, Qingdao, China. There were 269 clinical samples of throat and anal swabs from ducks and geese collected from 11 regions including Anhui, Fujian, Guangdong, Guangxi, Henan, Hubei, Hunan, Jiangsu, Jiangxi, Shandong and Sichuan.

### 2.2 Primer and probe design

The Mega 7 software was used to compare the genomes of DAstV-3, DAstV-4, GoAstV-1 and GoAstV-2, respectively, to identify the conserved regions of the virus. The primer and probe were designed with reference to the sequence of DAstV-3 (accession number: KJ020899), DAstV-4 (accession number: JX624774), GoAstV-1 (accession number: MH410610) and GoAstV-2 (accession number: ON745304). Primers and probes used for fluorescent RT-PCR were designed based on conserved sequence of gene using Oligo 7, and RT-PCR primers were designed to construct standard templates. To avoid the mutual interference between the emission spectra of fluorescent groups affecting the accuracy and specificity of the detection results of the established method, and considering the brand and model of the quantitative fluorescence PCR instrument, FAM, VIC, ROX and CY5 were used to label the fluorescent probes, respectively, in this study. Details of the final primers and exo-probes are shown in Table 1. All primers and exo-probes were synthesized by Sangon Biotech (Shanghai) Co., Ltd.

**Table 1:** Primers and probes used in this study.

Method	Primer probes	Sequences (5′—3′)	Target gene	Size (bp)
Quadruple fluorescent RT-PCR	DAstV-3-F	TGCGTACACCAACTAGAAAAC	ORF1b	144
	DAstV-3-R	TAGCAGCATACTCCTCAAC		
	DAstV-3-P	FAM-ATCCCAGCGTTCGTGAGTATCT-BHQ1		
	DAstV-4-F	GATGATGGAGAAGGCCCTTATGG	ORF2	82
	DAstV-4-R	TGTTGTTTCGCTACCCACCTGTC		
	DAstV-4-P	VIC-GCACCACACAACCGCTCGTCC-BHQ1		
	GoAstV-1-F	ACAACGTTTGAGTTTGGGTACTT	ORF1b	176
	GoAstV-1-R	AAGACATCGGCATACATCTTTA		
	GoAstV-1-P	ROX-GTTAAGTATATTTGCTATGGAGATGACAGGTT-BHQ2		
	GoAstV-2-F	CAGTGGACAACAATATGTGTAA	ORF1b	135
	GoAstV-2-R	CATCATCGCCATAGCAAATCA		
	GoAstV-2-P	CY5-GTCATTGCCGACGCTCAGATTA-BHQ2		
Sensitivity comparison RT-PCR primers	DAstV-3-F	TGAGTGTCCACGTTGTAAGCA	ORF1b	1,648
	DAstV-3-R	GCCATAATGCTACTTTCGGTC		
	DAstV-4-F	AGGCTGGAAGGGAATTCTATCT	ORF2	436
	DAstV-4-R	ATGGTGATTCCAGTGTCAGCG		
	GoAstV-1-F	AGTTGAGGGTTTGGAGGC	ORF1b	1,632
	GoAstV-1-R	CAGTGACCTTGTCGGCCATG		
	GoAstV-2-F	GTTCAAGAGTGTAGAGGAGCT	ORF1b	1,496
	GoAstV-2-R	ACACTATTGGGTGCATTTTCG		
ORF2 RT-PCR primers	DAstV-3 ORF2-F	AACAACCATGACCCAGAGACG	ORF2	2,297
	DAstV-3 ORF2 -R	AAGCGGGGCCAACACTAAAAG		
	DAstV-4 ORF2 -F	GGTAGGGAGGACCGAAATAAG	ORF2	2,174
	DAstV-4 ORF2 -R	GTTGTTTCGCTACCCACCTGT		
	GoAstV-1 ORF2 -F	GTAGAGGCCAATGTCAGGTAT	ORF2	1,372
	GoAstV-1 ORF2 -R	GTTAGTCACCTTGTCCACCCT		
	GoAstV-2 ORF2 -F	GGCACCACAAGTTCCCTATAC	ORF2	1,504
	GoAstV-2 ORF2 -R	AAGCCTAATGAGAAGGTGCAG		

### 2.3 Preparation of standard templates

The target gene fragment was amplified by RT-PCR using DAstV-3 ORF1bF/R, DAstV-4 ORF2F/R, GoAstV-1 ORF1bF/R, GoAstV-2 ORF1bF/R respective by HiScript High Fidelity One step RT-PCR Kit (Vazyme Biotech, China). The RT-PCR product was purified using a gel Recovery Kit (Thermo Fisher Scientific, China), then linked to the TA vector (Clone Smarter technologies, the United States of America) and transferred into *Escherichia coli* DH5α competent cells (Ruiboxingke biotechnology, China). Positive colonies were chosen for DNA sequencing identification after selection with ampicillincon LB plates. A plasmid Extraction Kit (Tiangen Biotech, China) was used to extract plasmids. Using Spe I incision enzyme to single enzyme of plasmids, and then the digested plasmid was purified by gel recovery kit (Takara Biotechnology, China). Using T7 (Yeasen Biotechnology, China) was transcribed kits for *in vitro* transcription, then using RNeasy Mini purification kits (QIAGEN Enterprise Management, China) to purification of transcription products to remove the impurity protein and various kinds of ions. The concentration of the recombinant plasmid was quantified using a NanoDrop 2000 spectrophotometer, and the copy number was calculated according to the formula: copies/μL = (6.02 × 10^23^) × ng/μL × 10^−9^/ (DNA length×660). Each plasmid was diluted from 1 × 10^7^ to 1 × 10^0^ copies/μL for use.

### 2.4 Condition optimization

The reaction condition of the quadruple fluorescence RT-PCR was optimized refer to requirements the Evo M-MLV One Step RT-qRT-PCR Kit II (Accurate Biotechnology, China), including the concentration of primers and probes, temperature of annealing.

### 2.5 Standard curve preparation

A quadruple fluorescence quantitative RT-PCR reaction system was configured, and the standard curves were generated and analyzed by using five gradients dilution standards of 1.0 × 10^6^~1.0 × 10^2^ copies/μL of four viruses as templates. RNase-free water was used as the negative control. All reactions were conducted in triplicates.

### 2.6 Specificity, sensitivity and reproducibility of quadruple fluorescence quantitative RT-PCR

The specificity of the quadruple fluorescence quantitative RT-PCR assay was evaluated based on cross-reactivity with other goose and duck viruses, includingDAstV-1 H9-AIV, NDV, ARV. In order to test the sensitivity of this method, four virus standards 1.0 × 10^7^~1.0 × 10^0^ diluted 10 times were used as templates, and the amplification was carried out according to the established quadruple fluorescence quantitative RT-PCR method, and compared with the standard RT-PCR method (Table 2). The repeatability experiment was carried in triplicate under optimized reaction conditions using three serial 10-fold diluted from 1.0 × 10^6^ copies/μL to 1.0 × 10^4^ copies/μL of standard, and the intra- and inter-group coefficient of variation (CV) was calculated for each assay.

**Table 2:** Intra- and inter-assay reproducibility of the of the quadruple fluorescent RT-PCR assay.

Name	Number of standard templates (copies/μL)	Intra-assay			Inter-assay		
		Mean	SD	CV (%)	Mean	SD	CV (%)
DAstV-3	1 × 10^6^	20.01	0.19	0.94	19.58	0.37	1.87
	1 × 10^5^	23.32	0.04	0.15	22.93	0.50	2.16
	1 × 10^4^	25.66	0.09	0.36	25.87	0.53	2.05
DAstV-4	1 × 10^6^	17.64	0.12	0.66	17.85	0.19	1.05
	1 × 10^5^	21.33	0.14	0.67	21.31	0.18	0.83
	1 × 10^4^	25.07	0.09	0.37	24.77	0.26	1.05
GoAstV-1	1 × 10^6^	16.45	0.23	1.41	16.82	0.44	2.64
	1 × 10^5^	20.19	0.22	1.08	20.47	0.25	1.20
	1 × 10^4^	23.37	0.06	0.25	24.17	0.67	2.77
GoAstV-2	1 × 10^6^	16.18	0.07	0.44	16.20	0.27	1.67
	1 × 10^5^	19.24	0.19	0.98	19.27	0.37	1.91
	1 × 10^4^	22.85	0.04	0.19	23.20	0.54	2.34

### 2.7 Clinical sample detection

To further verify the clinical potential of this quadruple fluorescence quantitative RT-PCR method assay, we tested 269 clinical samples from waterfowl. ORF2 sequence amplification was performed on the positive samples detected by multiplex fluorescence quantitative RT-PCR, and the primers for ORF2 amplification were established by our laboratory (Table 1).

## 3 Results

### 3.1 Quadruple fluorescence quantitative RT-PCR

To develop a quadruple fluorescence quantitative RT-PCR assay, different fluorescent-labeled target probes (DAstV-3 gene: FAM, DAstV-4 gene: VIC, GoAstV-1 gene: ROX, and GoAstV-2 gene: CY5) were first used. The concentration of recombinant plasmids was measured by NanoDrop 2000 spectrophotometer and the copy number of each virus plasmid was calculated. The initial concentration of virus was: 2.9 × 10^9^ for DAstV-3, 4 × 10^10^ for DAstV-4, 2.6 × 10^10^ for GoAstV-1, 4.6 × 10^10^ for GoAstV-2. Based on the single fluorescence quantitative RT-PCR method, the quadruple fluorescence quantitative RT-PCR method was established, and the reaction system and annealing temperature were optimized by the quadruple fluorescence quantitative RT-PCR method. The results of the optimal reaction system was determined by optimization as follows: 10 μL 2× One step RT-PCR Buffer II, 0.5 μL Pro Taq HS DNA Polymerase, 0.5 μL Evo M-MLV RTase Enzyme Mix II, the primers (10 μmol/L) of DAstV-3, DAstV-4, GoAstV-1 and GoAstV-2 are 0.8 μL, 0.8 μL, 0.6 μL and 0.6 μL respectively, and the probes (10 μmol/L) are 0.8, 0.6, 0.8, and 0.6 μL, respectively. Finally, 2.0 μL of RNA template and RNase-free water were added to a final volume of 25 μL. All reactions were performed on a QuantStudio™ 5 Real-time fluorescence quantitative PCR instrument (Thermo Fisher Technologies, Shanghai, China). The detection program was as follows: 42°C for 5 min and 95°C 30s, followed by 40 cycles of 95°C for 5 s and 60°C 30s.

### 3.2 Standard curve for the quadruple fluorescence quantitative RT-PCR assay

**Figure 1:**
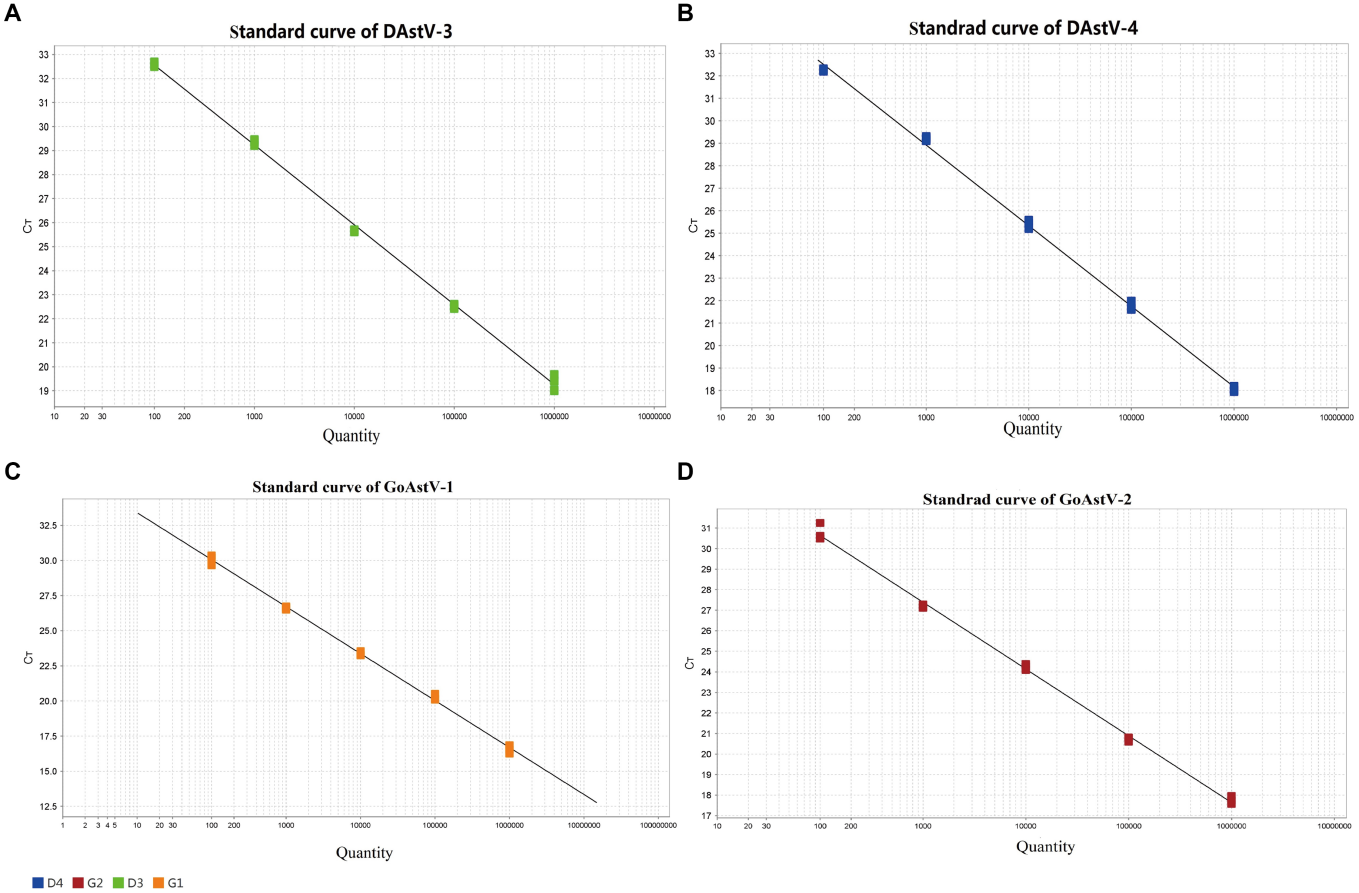
Establishment of a standard curve for the quadruple fluorescent quantitative RT-PCR assay. **(A)** Standard curve for DAstV-3; **(B)** Standard curve for DAstV-4; **(C)** Standard curve for GoAstV-1; (D) Standard curve for GoAstV-2.

Quadruple fluorescence quantitative RT-PCR was performed using 1.0 × 10^6^ ~ 1.0 × 10^2^ copies/μL of standards. The standard curves for DAstV-3, DAstV-4, GoAstV-1 and GoAstV-2 were drawn by plotting threshold cycle (Ct) on the x-axis and the log of the starting quantity on the y-axis. The standard equation are as follows (Figure 1): Y = −3.327Χ + 39,218, R^2^ = 0.998 (DAstV-3); Y = −3.587Χ + 39.685, R^2^ = 0.998 (DAstV-4); Y = −3.21Χ + 36.328, R^2^ = 0.998 (GoAstV-1); Y = −3.25Χ + 37.145, R^2^ = 0.997 (GoAstV-2).

### 3.3 Specificity of the quadruple fluorescence quantitative RT-PCR assay

**Figure 2:**
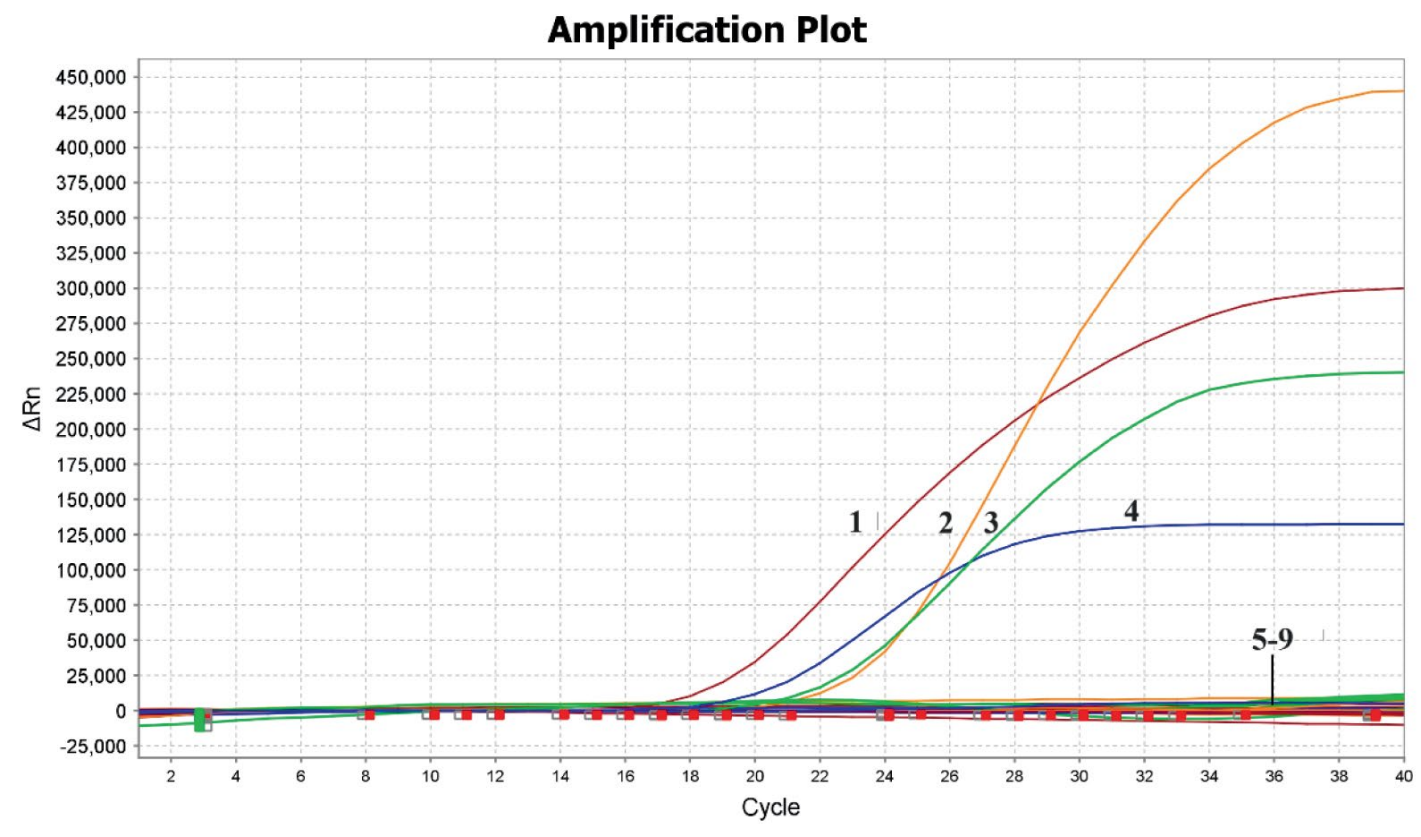
Specificity of the quadruple fluorescent quantitative RT-PCR assay. 1, GoAstV-2; 2, GoAstV-1; 3, DAstV-3; 4, DAstV-4; 5–9, DAstV-1, H9-AIV, NDV, ARV, Negative controls.

The results showed that positive for the nucleic acids of DAstV-3, DAstV-4, GoAstV-1 and GoAstV-2, and negative for the DAstV-1, H9-AIV, NDV, ARV and Negative controls. The developed multiplex RT-PCR assay demonstrated high specificity and did not cross-react with other viruses tested (Figure 2).

### 3.4 Sensitivity of the quadruple fluorescence quantitative RT-PCR assay

**Figure 3:**
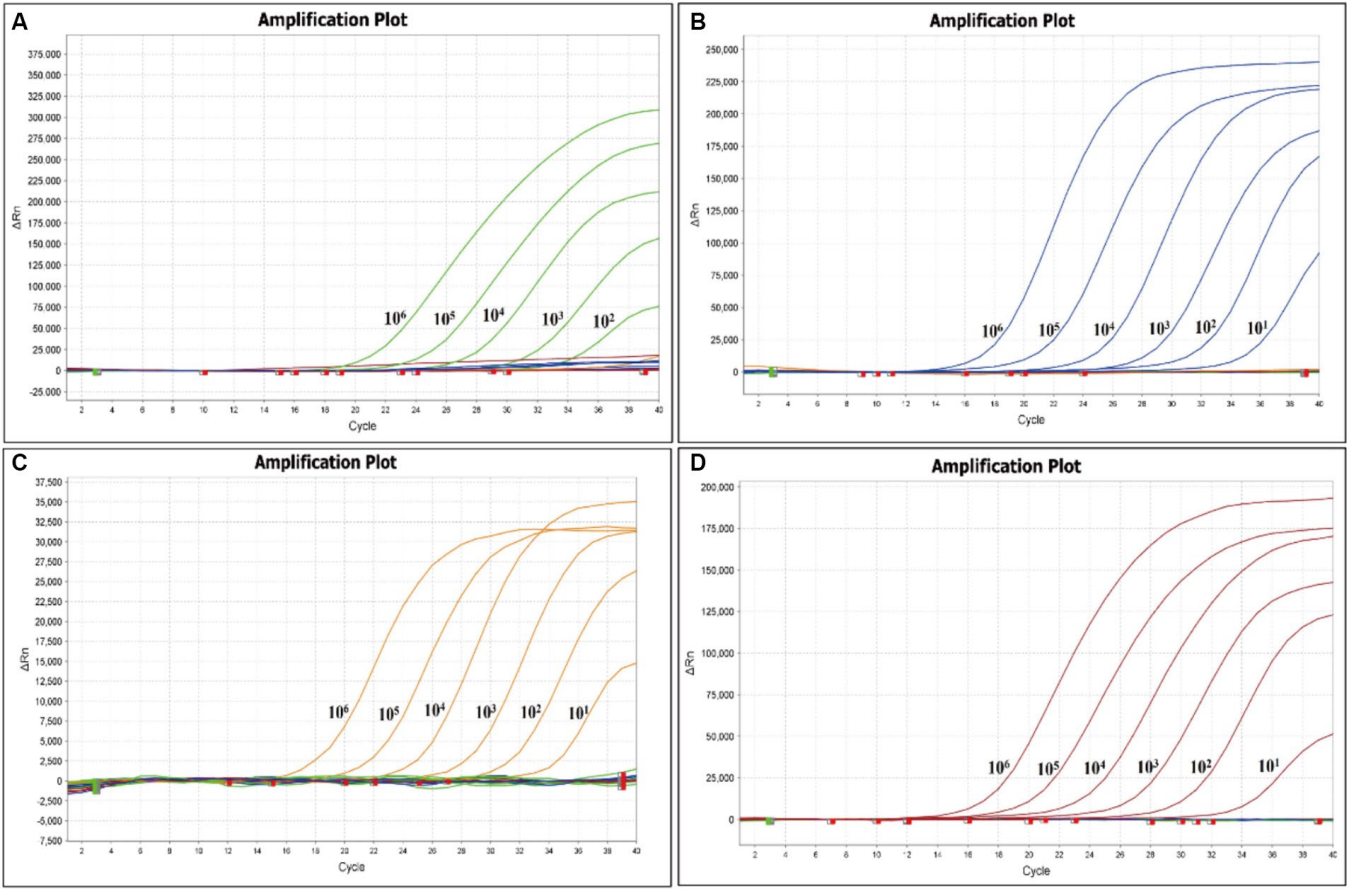
Sensitivity of the quadruple fluorescent quantitative RT-PCR assay. **(A)** Amplification plot for the DAstV-3; **(B)** Amplification plot for the DAstV-4; **(C)** Amplification plot for the GoAstV-1; **(D)** Amplification plot for the GoAstV-2.

**Figure 4:**
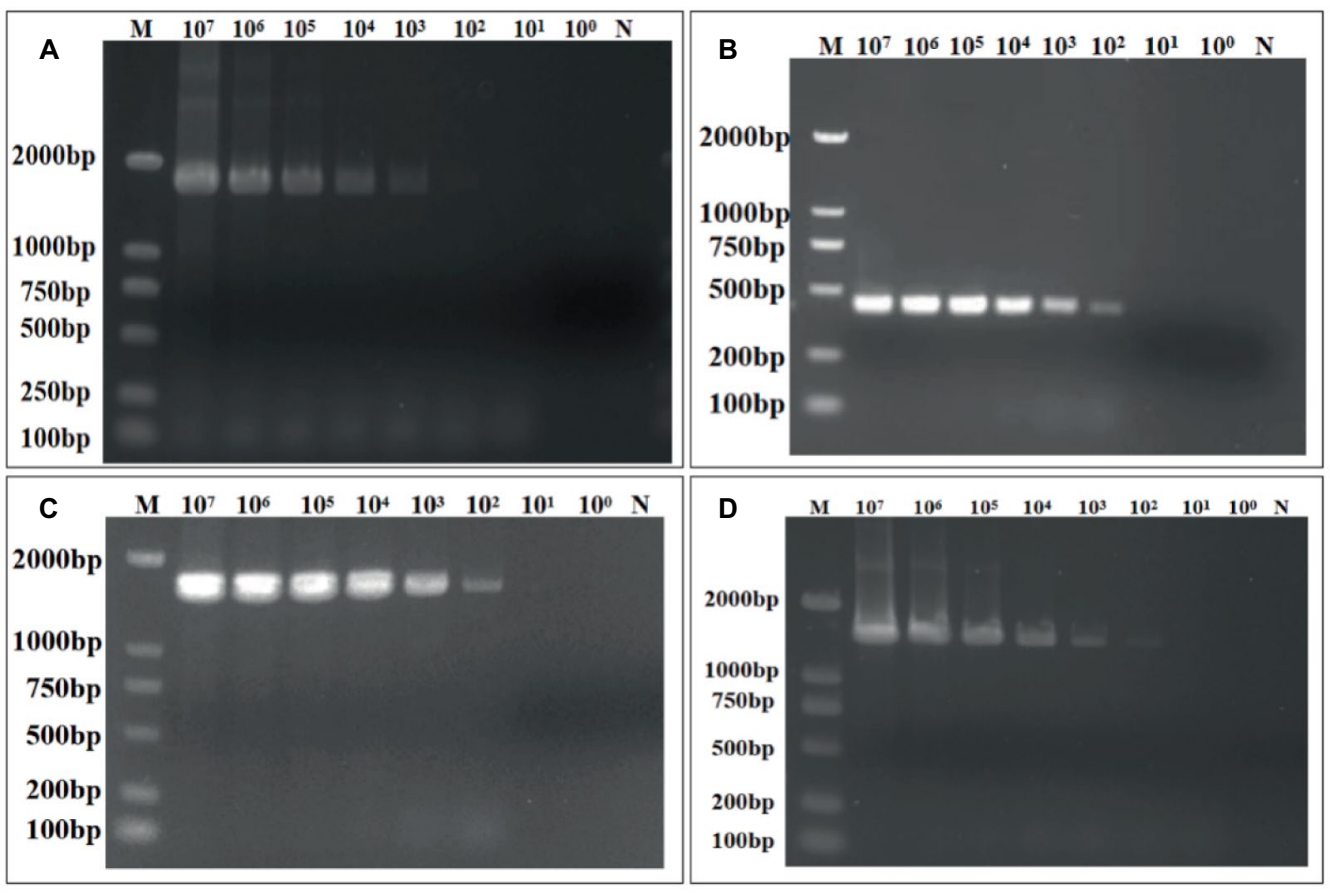
Sensitivity of the conventional RT-RT-PCR assay for the detection of DAstV-3, DAstV-4, GoAstV-1 and GoAstV-2. **(A)** DAstV-3 ORF1b; **(B)** DAstV-4 ORF2; **(C)** GoAstV-1 ORF1b; **(D)** GoAstV-2 ORF1b.

The sensitivity of the quadruple fluorescence quantitative RT-PCR assay was tested using serial 10-fold dilutions of the constructed four standard samples of waterfowl astrovirus. The results showed that this method has good sensitivity, while the sensitivity of DAstV-4, GoAstV-1 and GoAstV-2 was 10^1^ copies/μL, respectively, and the sensitivity of DAstV-3 was 10^2^ copies/μL (Figure 3). Whereas the sensitivity of the standard RT-PCR assay for DAstV-3 is 1 × 10^3^ copies/μL and that of DAstV-4, GoAstV-1, GoAstV-2 is 1 × 10^2^ copies/μL, respectively (Figure 4). Therefore, the sensitivity of the new method was 10 times higher than those of the conventional assay, respectively.

### 3.5 Repeatability and reproducibility of the quadruple fluorescence quantitative RT-PCR assay

The intra- and inter-assay CVs were evaluated at concentrations of 1.0 × 10^6^, 1.0 × 10^5^, 1.0 × 10^4^ copies/μL of standard, respectively. The intra-assay CV. was <1.5% and the inter-assay CV was <3%, indicating that the quadruple fluorescence quantitative RT-PCR assay is repeatable and reproducible (Table 2).

### 3.6 Clinical sample detection

Based on the above results of sensitivity, we next sought to verify the capability of this quadruple fluorescence quantitative RT-PCR assay to detect clinical samples. As shown in Table 3, we tested to detect these viruses in clinical samples, 15.3% were found to be positive for DAstV-4, 1.6% for DAstV-3, 1.6% for GoAstV-1 and 0.7% for GoAstV-2. Besides, we also identified the presence of co-infection of DAstV-4 with GoAstV-1 and DAstV-4 with GoAstV-2, respectively. Sequencing was performed on RT-PCR products of the ORF2 gene, which verified the results of the quadruple fluorescence quantitative RT-PCR assay.

**Table 3:** Positive rates (%) of four types of astrovirus.

Waterfowl-derived astrovirus	Positive number	Positive rate (positive number/total number)
DAstV-3	4	1.6% (4/269)
DAstV-4	41	15.3% (41/269)
GoAstV-1	4	1.6% (4/269)
GoAstV-2	2	0.7% (2/269)
DAstV-4 + GoAstV-1	3	1.1% (3/269)
DAstV-4 + GoAstV-2	1	0.4% (1/269)
Total	55	20.4% (55/269)

## 4 Discussion

DAstV-3, DAstV-4, GoAstV-1 and GoAstV-2 as newly discovered waterfowl-derived astrovirus that have caused reduced production performance and even death of ducks and geese in recent years, there is no clear classification. To control the disease, it is important to establish a reliable, it is important to establish a reliable, rapid, accurate, and sensitive method for the detection of DAstV-3, 4 and GoAstV-1, 2. At present, the main laboratory detection methods for avian astrovirus are molecular biological diagnostic techniques. Among them, fluorescence quantitative RT-PCR detection method is widely used in laboratory detection because of its short time consumption and high sensitivity. In previous studies, a TaqMan-based quantitative RT-PCR method was established for the detection of GoAstV-2 alone and a duplex RT-PCR method to simultaneously detect and differentiate between GoAstV-1 and GAstV-2 (Yi et al., 2022). However, it is more practical to develop a quadruplex quantitative RT-PCR method to simultaneously detect and discriminate between DAstV-3,4 and GoAstV-1,2 in field clinical samples.

The genome structure of AAstV consists of three open reading frames, among which ORF1b encodes RDRP, which is a conserved sequence of the virus. ORF2 encodes virus coat protein, which can react specifically with virus-specific antibodies and help to determine the phylogenetic relationship between viruses. In this study, in order to achieve rapid detection of DAstV-3, DAstV-4, GoAstV-1 and GoAstV-2, initially selected four conserved genes of waterfowl astroviruses, ORF1b, and designed specific primers and probes. However, when the primers and probes of DAstV-4 were in the ORF1b gene region, The specificity and sensitivity effects were not ideal, so attempts were made to design primer probes in the ORF2 region that determines the typing of avian astrovirus, which could cooperate with the other three waterfowl-derived astrovirus primer probes without cross-reaction. Therefore, in this study, a quadruple fluorescent quantitative RT-PCR method was established for the highly conserved ORF1b region of DAstV-3, GoAstV-1 and GoAstV-2 and the ORF2 region of DAstV-4. The method had good specificity and had no cross-reaction with other avian viruses. The sensitivity of the quadruple fluorescence quantitative RT-PCR for DAstV-3, 4 and GoAstV-1, 2 were 10 times higher than those of the conventional assay, respectively. The intra-assay and inter-assay coefficients of variation were less than 3%, suggesting good reproducibility.

The results of clinical samples showed that the positive rate of DAstV-4 was the highest, with 15.3%, that of DAstV-3 and GoAstV-1 was 1.6%, and that of GoAstV-2 was 0.7%. There are co-infections between DAstV-4 and GoAstV-1 and between DAstV-4 and GoAstV-2, but the specific infection and transmission mechanism need to be studied. In order to further verify the method, ORF2 amplification was carried out on the samples which were positive by quadruple fluorescence quantitative detection, and the amplified products were sent to sequencing. The RT-PCR products were sequenced and the results confirmed that quadruple fluorescence quantitative RT-PCR assay was reliable.

In summary, a quadruple fluorescent quantitative RT-PCR method was successfully established in this study. The assay proved to be specific, sensitive, reliable, and feasible for testing field clinical samples. This newly developed assay represents a useful tool for the diagnosis and detection of DAstV-3, 4 and GoAstV-1, 2, and their co-infection, and should facilitate molecular epidemiological investigations of these viruses.

## Data availability statement

The original contributions presented in the study are included in the article/supplementary material, further inquiries can be directed to the corresponding authors.

## Ethics statement

The animal studies were approved by Animal Welfare Committee of the China Animal Health and Epidemiology Center; It is attributed to China Animal Health and Epidemiology Center. The studies were conducted in accordance with the local legislation and institutional requirements. Written informed consent was obtained from the owners for the participation of their animals in this study.

## Author contributions

YL: Funding acquisition, Writing – original draft, Writing – review & editing. JL: Methodology, Writing – original draft, Writing – review & editing. FZ: Data curation, Writing – review & editing. JS: Investigation, Writing – review & editing. CD: Conceptualization, Investigation, Writing – review & editing. YF: Investigation, Writing – review & editing. GM: Validation, Writing – review & editing. WJ: Methodology, Writing – review & editing. XY: Data curation, Writing – review & editing. GL: Data curation, Writing – review & editing. HL: Funding acquisition, Resources, Writing – original draft, Writing – review & editing.
